# Microglia and Astrocyte Function and Communication: What Do We Know in Humans?

**DOI:** 10.3389/fnins.2022.824888

**Published:** 2022-02-16

**Authors:** Emma F. Garland, Iain J. Hartnell, Delphine Boche

**Affiliations:** Clinical Neurosciences, Clinical and Experimental Sciences, Faculty of Medicine, University of Southampton, Southampton, United Kingdom

**Keywords:** microglia, astrocytes, Alzheimer’s disease, human, neuroinflmamation, genetics, biomarkers

## Abstract

Microglia and astrocytes play essential roles in the central nervous system contributing to many functions including homeostasis, immune response, blood–brain barrier maintenance and synaptic support. Evidence has emerged from experimental models of glial communication that microglia and astrocytes influence and coordinate each other and their effects on the brain environment. However, due to the difference in glial cells between humans and rodents, it is essential to confirm the relevance of these findings in human brains. Here, we aim to review the current knowledge on microglia-astrocyte crosstalk in humans, exploring novel methodological techniques used in health and disease conditions. This will include an in-depth look at cell culture and iPSCs, *post-mortem* studies, imaging and fluid biomarkers, genetics and transcriptomic data. In this review, we will discuss the advantages and limitations of these methods, highlighting the understanding these methods have brought the field on these cells communicative abilities, and the knowledge gaps that remain.

## Introduction

**FIGURE 1:**
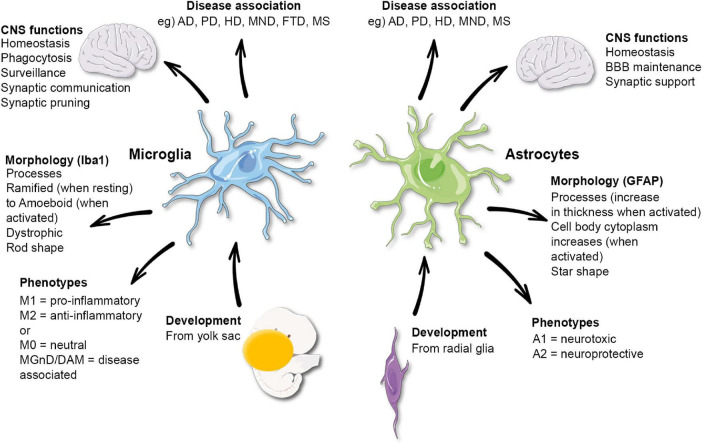
Functional and morphological characteristics of microglia and astrocytes. AD, Alzheimer’s disease; PD, Parkinson’s disease; HD, Huntington’s disease; MND, motor neuron disease; FTD, frontotemporal dementia; MS, multiple sclerosis. Microglia phenotypes have been disputed and may not reflect their functions.

Astrocytes and microglia, both types of glial cells, are key cells in the central nervous system (CNS), maintaining homeostasis and supporting the function of neurons (Figure 1). The communication between neurons and the glial cells is well recognized. However, crosstalk between astrocytes and microglia is also an essential feature of a healthy CNS, and the breakdown of this communication may be an important mechanism in neurodegenerative disorders. The study of glial communication in humans remains a challenge due to the complexity, access, and function of the CNS. However, tools have been developed in an attempt to overcome these difficulties, with some of them specifically designed to explore glial cells.

Here we review examples in the literature of the current understanding of microglia and astrocytes functions and their communication in the human brain that has been gained using some of these tools, with a focus on Alzheimer’s disease (AD) and other neurodegenerative diseases.

The research discussed here will include genetic and transcriptomic studies which evaluate genes and proteins expressed by glia and their relevance to disease. The functional studies reviewed look at cultured human cells using primary isolated glial cells, or cells derived from human induced pluripotent stem cells (iPSC). The stem cell model systems have been generated to closely mimic the human environment whilst allowing cellular manipulation to improve our understanding of their functions. For these characteristics, iPSCs have become the most utilized cell system in neurodegenerative disease (Table 1), used individually or in co- or tri-culture systems to investigate cell cooperation and coordination in what is known as the cerebral organoid.

**TABLE 1:** Current methods available to study human glial cells.

Method	Advantages	Disadvantages
Genetics	Identifies candidate genes and pathways relevant to disease.	Requires confirmation of the cellular/biological implications.

Transcriptomics	Identify specific cell-type pathways.	Very large amount of data obtained.
		Lack of consistency based on the source of the cells and technique used.

Primary culture	Can study human cells independently of their environment.	Cells may behave differently to *in vivo*.
	Allows easy control of the cell environment.	Challenging to obtain from humans.

iPSCs	Minimally invasive.	Derived cells may be different from astrocytes/microglia *in vivo.*
	Sources directly from patients.	Non -cerebral origin.
	Retains some of the human specificities after re-differentiation.	Challenging to maintain overtime.

*Post-mortem*	Study of glial cells *in situ.*	Provides a static, late-stage picture rather than dynamic image of the events.
	Identification of several cell populations.	Post-mortem delay
		Tissue preservation for some methods.
		High heterogeneity of the human population

PET	Real time functional readout for a specific cell type.	Low cell specificity.
	Moderately invasive method.	TSPO polymorphism affects binding.
	Many ligands being developed.	Requires injection of radioactive tracer. Expensive.

MRI	Real time readout.	Absence of cell specificity.
	Non-invasive.	

Biomarkers	Temporal investigation.	May not reflect brain inflammation.
	Indicator of inflammatory status.	Can be invasive for patients (lumbar puncture).
	Potential for predictive readout.	Link with glial cells unclear.
	Can follow therapeutic effects *in vivo.*	

Other studies have explored astrocytes and microglia *in situ* in human brain tissue, through *post-mortem* immunohistochemical studies to examine cells at a specific time in their real environment. Finally, recent non-invasive techniques will be also discussed which assess microglia/astrocyte activation in patients during life, allowing temporal investigation of their activated status via the use of brain imaging and fluid biomarkers (Table 1).

## An Overview

### Microglia

Microglia are the resident immune cells of the CNS, accounting for 10% of cells (Salter and Stevens, 2017). They are derived from the yolk sac (Ginhoux et al., 2010), and during brain development regulate neurogenesis (Cunningham et al., 2013), promote neuronal survival (Ueno et al., 2013) and participate in synaptic pruning, to ensure appropriate neuronal connections are made and brain maturation occurs (Schafer et al., 2012). Throughout adult life, they continue to interact with their environment, contributing to synaptic communication and ensuring cerebral homeostasis is maintained within the brain by constantly surveying the surrounding parenchyma with their finger-like processes (Boche et al., 2013). They have recently been defined as a multifunctional housekeeping cell type (Cserép et al., 2021). However, the exact function of microglia is still somewhat unclear in human neurodegenerative disease, but evidence supports a role for these cells in conditions such as AD (Franco-Bocanegra et al., 2019b), Parkinson’s disease (PD) (Lecours et al., 2018), and frontotemporal dementia (FTD) (Hartnell et al., 2021).

Microglia have been considered to exist in two states referred to as ‘resting’ or ‘activated.’ ‘Resting’ microglia were classed as those that are highly ramified in their morphology with processes allowing them to scan the microenvironment for foreign bodies and neuronal injury (Boche et al., 2013). The term ‘resting’ was not accurate as the ramified microglia are highly dynamic cells, as observed in the rodent brain (Nimmerjahn et al., 2005). In the context of cerebral homeostatic changes (e.g., due to infection, damage, or disease), the cells become ‘activated’ and undergo morphological changes that include enlarged cell bodies with thickening and shortening of their processes, leading to a more amoeboid-like morphology and migration toward the “anomaly” (Boche et al., 2013; Franco-Bocanegra et al., 2021). In addition, the morphological modifications are associated with the expression of inflammatory molecules in response to the homeostatic disturbance of the micro-environment (Hanisch, 2002). Two types of microglial activation status were initially considered and named M1 and M2. M1 was referred to as *classic activation* where the cells expressed pro-inflammatory cytokines, targeted foreign bodies, exhibiting antigen presentation markers and performing phagocytosis (Mosser and Edwards, 2008). The M2 status was known as *alternate activation/wound healing*. Again, microglia in the M2 profile performed phagocytosis but also promoted changes in the extracellular matrix as a healing process and the profile was associated with the expression of anti-inflammatory cytokines (Edwards et al., 2006). This categorization, which was derived from the macrophages in experimental conditions, is now disputed. Indeed, several microglial populations have been detected cohabiting within the same brain, with specific disease-associated microglial profiles identified in experimental models (Boche and Gordon, 2021) and conditions such as AD, associated with a mix of pro and anti-inflammatory molecules expressed in the brain (Rakic et al., 2018; Franco-Bocanegra et al., 2019a). However, consensus on the number and functions of microglial populations and the presence of disease-specific profiles as identified in models remains to be established in humans.

### Astrocytes

Astrocytes are a subset of glial cells which contribute to the maintenance and regulation of neuronal function. These star-shaped cells make up between 17 and 61% of the cells in the human brain, depending on the area (von Bartheld et al., 2016). Astrocytes have several functions within the healthy CNS including neurotransmitter cycling, metabolic support of neurons and maintenance of the blood–brain barrier (via their roles in neuro-vascular coupling) (Sofroniew and Vinters, 2010).

**FIGURE 2:**
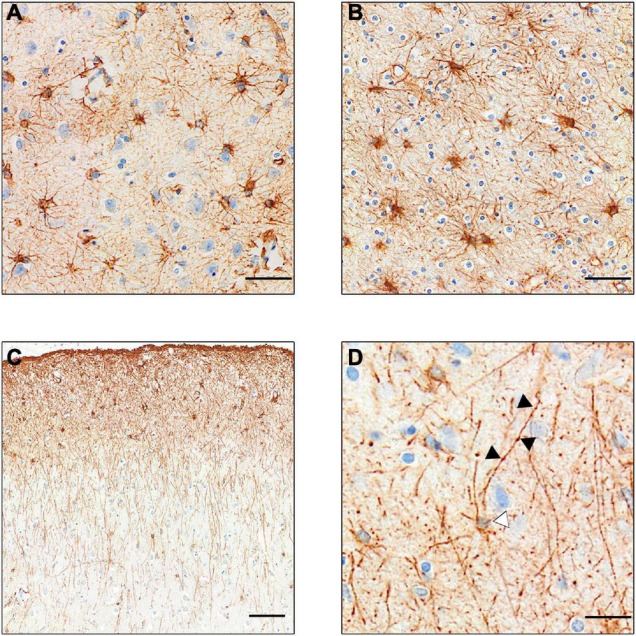
GFAP+ stained astrocyte subtypes. **(A)** Protoplasmic astrocytes of the gray matter. Note the lighter staining, showing less expression of GFAP. **(B)** Darker stained fibrous astrocytes of the white matter. **(C)** Intralaminar astrocytes stretching process from layer I–II to layers III and IV. **(D)** Varicose projection astrocyte. Black arrows point to varicosities on the straight primary process. White arrow points to faintly stained cell body. [Scale bar **(A,B)** = 50 μm, **(C)** = 100 μm, **(D)** = 25 μm].

Our current knowledge of astrocytes is mostly sourced from studies in rodents; however, as with microglia, human astrocytes differ quite markedly from those of rodents. For example, differences were observed in calcium signaling functions and transcriptome readouts between human and mouse astrocytes (Zhang et al., 2016). There are several subclasses of astrocytes (see Figure 2) the most numerous of which are protoplasmic astrocytes, found in all mammals. These have a stellate morphology and inhabit their own distinct domain in layers II–VI of the gray matter never overlapping other astrocytes. However, in humans, these astrocytes are three times larger with more than ten times the number of projections than those of rodents (Oberheim et al., 2009). There are also two distinct subtypes of astrocytes found only in primates and humans which reside in either layer I or layer VI and reach projections down (or up) into the other cortical layers (Oberheim et al., 2006). These interlaminar astrocytes have high expression of CD44, GFAP (glial fibrillary astrocytic protein) and S100B (S100 calcium-binding protein B), but low expression of glutamate processing markers excitatory amino acid transporter (EAAT)- 1 and EAAT2 and Glutamate Synthetase (Sosunov et al., 2014). Similar transcriptional properties were also seen in the white matter “fibrous astrocytes.”

**FIGURE 3:**
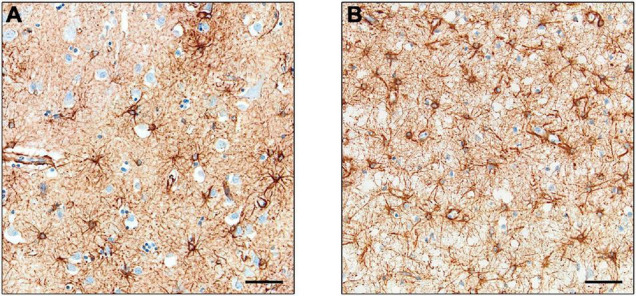
Expression of GFAP+ astrocytes. Astrocytes in control cases **(A)** and Pick’s disease cases **(B)**, with the latter showing darker staining and greater cell number. Scale bars = 50 μm.

Interest has increased in the role of astrocytes during disease, particularly in their participation in the neuroinflammatory processes. Changes in astrocyte morphology, function and abundance (termed “astrogliosis”) have been observed in human neurodegenerative disorders (Pekny and Pekna, 2014). However, many studies used GFAP to monitor astrocyte proliferation, and it is argued that the increased expression may be related to upregulation in individual cells rather than astrocytic proliferation (Perez-Nievas and Serrano-Pozo, 2018), as observed in a study using the proliferating cell marker PCNA (Marlatt et al., 2014). Furthermore, studies using cell counts of astrocytes – through Nissl staining (Pelvig et al., 2003) or constitutive astrocyte markers in combination with GFAP (Serrano-Pozo et al., 2013a) – showed no difference in cell number in AD compared to control brains. Examples of GFAP in a control brain and a brain with Pick’s disease are shown in Figure 3.

Further to their increased GFAP expression during neuroinflammation, astrocytes take on a neurotoxic phenotype (A1) (Zamanian et al., 2012) which loses neuroprotective abilities, leading to neuronal death. This A1 phenotype was revealed in AD, Huntington’s disease (HD), motor neuron disease (MND), and PD (Liddelow et al., 2017). However, the idea that astrocytes become polarized to an A1 (neurotoxic) or A2 (neuroprotective) phenotype upon activation from different signals issued from microglia may be misleading. As with the previous M1/M2 distinction in microglia (from which the field has moved on) (Ransohoff, 2016), it seems that despite the discovery of a 12 gene signature of A1 astrocytes (Liddelow et al., 2017), intermediate or divergent transcriptomic states might exist and even co-exist within the same brain (Escartin et al., 2021). Other studies have also cast doubt on the polarization of the astrocytic phenotype, showing that the transcriptomic signature of disease-related astrocytes in AD (Grubman et al., 2019; Zhou et al., 2020) or HD (Al-Dalahmah et al., 2020) did not overlap with the one initially described by Liddelow et al. (2017).

A phagocytic role for astrocytes has also be identified in the humans. In AD, reactive astrocytes via their processes are known to form clusters around amyloid plaques and APP-containing dystrophic neurites (Gomez-Arboledas et al., 2018). However, the number of astrocyte processes correlated with the amount of plaque-associated neurites rather than plaque size, suggesting that they are responding to neuronal damage rather than the plaques *per se* (Serrano-Pozo et al., 2013b). Using immunofluorescence, it was shown that GFAP+ processes closely contact APP-containing neurites. Using transmission electron microscopy, they observed that the astrocytes-neurites association involved astrocytic phagocytosis with dystrophic neurites internalized by reactive astrocytes (Gomez-Arboledas et al., 2018). This supports that a dysfunction in astrocytes’ ability to carry out phagocytosis may contribute to the progression of AD.

## Genetics and Transcriptomic Studies

### Microglia

Genetic data has been invaluable in providing a greater understanding of microglia and their function in neurological conditions. Being derived from the early erythro-myeloid progenitors in the yolk sac (Ginhoux et al., 2010), many genes contribute to their differentiation and role as immune cells within the CNS (Wurm et al., 2021). As aging occurs, microglial gene expression changes and this might potentially explain why aging is the major contributor to disease risk in neurodegenerative conditions (Olah et al., 2020).

Genome-wide association studies (GWAS) allow the identification of inherited genetic variants associated with disease risk by testing an extremely large data set for important genetic information. It was well established that the *APOE* (apolipoprotein E) genotype was, with aging, an important risk factor in developing AD (Saunders et al., 1993). There is a protective APOE allele (ε2), a neutral allele (ε3), and a risk allele (ε4). An individual who is homozygous for the ε4 allele has an 8-12 times greater chance of developing AD (Genin et al., 2011; Serrano-Pozo et al., 2015). Twenty years later, one GWAS study identified 11 novel genes associated with the risk of AD. These included *HLA-DRB5–DRB1, SORL1, PTK2B, SLC24A4, ZCWPW1, CELF1*, *CASS4*, and *FERMT2* genes (Lambert et al., 2013), highlighting the involvement of several immune genes. Other GWAS studies have then focused on the association between microglial genes and AD and found that *TREM2, CD33, CR1*, and *PLGC2* were all significant risk factors for the disease and that they may alter the functionality of microglia (Malik et al., 2015). Of note, from all GWAS studies in AD, the *APOE* gene was still the strongest risk factor identified for this disease. Triggering receptor expressed on myeloid cells 2 (TREM2) is of particular interest as the identified genetic variant confers the same magnitude of genetic risk as *APOE* (Guerreiro et al., 2013; Jonsson et al., 2013). However, the *TREM2* variant is present in 1% of the population when the *APOE* ε4 genotype associated with AD is expressed by 30% of the population. There is evidence for upregulation of TREM2 in AD (Perez et al., 2017), but its exact role in this disease and its presence in human microglia remains to be confirmed (Fahrenhold et al., 2018). GWAS studies are crucial for discovering genetic risk factors linked to disease, but they do not show the whole picture of which protein, cell or process is involved. Therefore, to determine the function and role of the gene/protein in particular circumstances, other tools and techniques are usually necessary.

The development of the single-cell RNA sequencing (scRNAseq) method has allowed the identification of different microglial populations based on genetic expression. This is a high-throughput technology (Svensson et al., 2018) that can distinguish between microglia and other myeloid cell populations (e.g., perivascular macrophages) using specific microglial proteins such as the transmembrane protein 119 (TMEM119) (Bennett et al., 2016). This method, in both mouse models and human brain samples, has re-emphasized that microglial genes are highly associated with neurodegenerative disease (Keren-Shaul et al., 2017; Olah et al., 2020). A study using scRNAseq on human brain tissue including mild cognitive impairment (MCI), AD or epilepsy patients, identified 9 different microglial subpopulations with different functionalities, including homeostasis, antigen presentation and proliferation. Four of these subpopulations were similar to the disease-associated microglia transcriptomic profiles defined in experimental models (Olah et al., 2020). Another human study identified seven different populations and did not find a difference in the gene expression profiles of microglia, suggesting an absence of specific AD-related microglial populations (Alsema et al., 2020). The discrepancy between studies emphasizes the challenges associated with this technology when used in the human brain (Boche and Gordon, 2021), partly due to the small number of microglia and the need to pool several brains together leading to conditions being mixed. It also highlights the need to confirm the experimental findings in humans to advance our understanding of microglia.

Single-cell RNA sequencing has more recently challenged the microglia activation classification groups, M1 and M2, with results showing that these may be an oversimplification of the roles of microglia. Evidence suggests a progressive switch in gene expression that causes microglia to become activated, rather than a distinct on or off mechanism. Also, it has been shown that the progressive switch may be related to the proximity of Aβ plaques in the context of AD (Keren-Shaul et al., 2017). In experimental models, transcriptomics identified two main gene clusters associated with microglia, these being a homeostatic cluster given the term ‘M0,’ defined by *P2ry12, Tmem119, Cx3Cr1*, and an inflammatory cluster termed ‘MGnD’ defined by *Ccl2, Csf1*, and *Apoe*. The same study also reported that TREM2 induces the APOE pathway which then causes a switch between M0 and MGnD microglial phenotypes, and thus targeting this pathway could reinstate the homeostatic nature of microglia in AD (Krasemann et al., 2017). Some of the microglial phenotypes described from mouse brains have been confirmed in humans (Boche and Gordon, 2021).

Lastly, single nucleus RNA sequencing (snRNAseq) has been developed as an alternative method to scRNAseq to explore transcriptomics in tissue where it is difficult to extract cells and keep them intact. It also has the advantage of being performed on frozen rather than fresh tissue and thus can make use of archived, well-characterized cases. Using this methodology on two brains, a total of five microglial clusters were identified with the presence of microglia genes highly expressed in some of the clusters such as the homeostatic gene *P2Y12* in and *C1QA*. The authors reported similar findings on microglia isolated from fresh tissue from the same donors and concluded this to be proof of the relevance of the snRNAseq method to study microglial genes (Gerrits et al., 2020). Indeed, nuclei isolated from human microglia can provide a substantial genetic map to investigate important microglial genes. However, this has been challenged by another study also comparing scRNAseq and snRNAseq on microglia isolated from frozen biopsies. They observed that genes associated with microglial activation identified in scRNAseq (e.g., *APOE*, *SPP1*, and *CD74*) were depleted in snRNAseq (Thrupp et al., 2020), implying a low sensitivity of the method and that microglial nuclei may not recapitulate the whole picture of microglial activation. The different studies clearly demonstrated the challenges associated with the study of microglia in humans.

### Astrocytes

As with microglia, *APOE* is also an important gene when it comes to astrocytes in the context of AD. Astrocytes are the primary APOE expressing cells of the brain. In humans, APOE ε4 carriers had similar numbers of GFAP+ astrocytes (either reactive or resting) to other APOE genotypes (Serrano-Pozo et al., 2011). Whilst similar in number, their function may be altered, as in astrocytes derived from human iPSCs, an APOE ε4/ε4 genotype was found to cause a loss of neurotrophic function (Zhao et al., 2017).

To uncover other genes in astrocytes that may be involved in the pathological process in AD, transcriptomic studies compared astrocyte genomes between control and AD brains. Laser capture microdissection was utilized to isolate astrocytes from *post-mortem* human tissue for their transcriptomes to be analyzed using gene microarrays. This technique has allowed the identification of several astrocytic genes including the mitochondrial genes *PITRM1-AS1* (pitrilysin metallopeptidase 1 antisense RNA1), an antisense copy of a gene known to degrade Aβ (Falkevall et al., 2006), *NDUFA4L2* (NADH dehydrogenase 1 alpha subcomplex, 4-like 2) which inhibits respiratory complex 1 in mitochondria, and *FASKD2* (fast kinase domain-containing protein 2) involved in regulating apoptosis. Other immune-related genes found to be different in AD astrocytes were *C3* (complement component 3), *CLU* (Clusterin) and *CD74* (cluster of differentiation 74) (Sekar et al., 2015). Another study also reported changes in genes across several functional pathways between early and late-stage AD. The main differences were detected between the Braak stages 1–II vs. Braak stages V–VI and were related to reductions in signaling pathways related to insulin, MAPK (mitogen-activated protein kinase), PI3K (phosphatidylinositol 3-kinase), and PKB (protein kinase B). Furthermore, they revealed that many pathways in astrocytes were also modified with respect to the *APOE* genotype (Simpson et al., 2011).

## Human Primary Cell Cultures and Induced Pluripotent Stem Cells

Cell culture has been a commonly used method to study glial cells and allows the control of the physiochemical environment of the cell. However, it was argued that the representation of the native/physiological environment of cells was limited in single-cell culture model systems though there are ways of mimicking *in vivo* systems in this *in vitro* method. Several glial cell lines are commercially available, some of them derived from human cells, but for cell lines to acquire the necessary properties to grow *in vitro*, there is a loss of their specificity.

The culture of primary glial cells has the advantage of being prepared from cells isolated directly from the brain and hence provide more relevant findings than cell lines. These cells are often sourced from rodent pups, and are not representative of differentiated/mature cells and thus require to be highly manipulated to mimic neurodegenerative diseases associated with aging. In addition, human microglia and astrocytes are known to be significantly distinct from those of the rodents (Oberheim et al., 2009; Franco Bocanegra et al., 2018). Therefore, primary human cell culture can provide avenues to explore human cell physiology in health and disease, although with similar limitations as for cell lines, namely its simplicity and disconnection from the original cell environment. Furthermore, primary cell culture from humans is a particular challenge, as it requires either fetal cells (that may not represent those in chronic disease), or cells collected from adults during neurosurgical operations (that might not be relevant to normal physiology or neurodegenerative diseases), or cells isolated from post-mortem brains. Overall, primary human cells are extremely challenging to obtain and involve complex ethical considerations.

Induced pluripotent stem cells are a more accessible model in which to study the behavior of human cells. Cells from the skin and blood (Lowry et al., 2008) are sourced from an individual and reprogrammed into embryonic stem cell-like cells (known as iPSC cells) using the Yamanaka factors and have been generated from a variety of differentiated cells (Takahashi and Yamanaka, 2006). iPSCs can be re-differentiated into any cell type and iPSC-based methods are now widely used to study the physiopathology of disease or as platforms in drug discovery with iPSCs banks being established.

### Microglial Cell Culture Models

Microglia derived from human iPSCs have been generated to mimic physiological microglia (sourced from healthy donors) or disease microglia (provided by patients). Microglial cells derived from healthy human iPSCs were able to phagocytose foreign particles, as previously observed in cell cultures, but most importantly expressed microglial-specific markers such as TMEM119. Furthermore, when microglial iPSCs were co-cultured with neurons, the microglial signature was enhanced and the microglial cells presented a ‘surveying behavior’ similar to the process in physiological conditions. Lastly, when damage was caused to the cell co-culture, the microglia reacted within minutes to these stimuli by extending their processes to the site of injury (Muffat et al., 2016), starting the inflammatory process as expected (Nimmerjahn et al., 2005; Franco-Bocanegra et al., 2019b). One study examined the consequences of the main AD risk factor APOE ε4 on microglia. Interestingly, microglia associated with this allele revealed a different morphology compared to those associated with the APOE ε3 allele. APOE ε4 microglia presented decreased process length and were fewer in number (Lin et al., 2018), which might be associated with decreased phagocytic capabilities, specifically phagocytosis of Aβ. Furthermore, with the use of the CRISPR/Cas9 system, iPSCs with the APOE ε4 allele were converted to the APOE ε3 allele and this consequently caused a reduction in AD pathology (Lin et al., 2018), suggesting that the role of APOE as a risk factor for AD might be triggered by microglia. Interestingly, a study observing the effect of *TREM2* mutations on microglia using human iPSCs, found that the missense mutations of this gene caused microglia derived from these donors to express less TREM2. This initiated a reduction in phagocytic capabilities of the human iPSC derived microglia, suggesting that *TREM2* missense mutations cause dysfunction in microglia (Garcia-Reitboeck et al., 2018). Furthermore, using iPSCs derived from individuals with a *TREM2* mutation that causes FTD, it was discovered that the mutant form of TREM2 could not be processed by microglia, staying in its immature form, and was not properly relocated to the plasma membrane of the cells. However, it was shown that the microglia with this *TREM2* mutation could still respond to inflammatory stimuli (Brownjohn et al., 2018). The recent combination of iPSCs based on identified risk factors has highlighted potential microglial mechanisms in AD and other dementias. One study was able to demonstrate that human iPSC microglia recapitulated the ability to phagocytose Aβ and tau both *in vivo* and *in vitro*. The cells were also able to release cytokines when exposed to inflammatory stimuli and underwent the same genetic changes from said stimuli as physiological microglia would behave in human brain (Abud et al., 2017; Garcia-Leon et al., 2020).

To attempt to closely recapitulate the microenvironment of the human brain, multiple cerebral cell types have been cultured together. One study developed a co-culture of embryonic iPSC-derived macrophages with iPSC-derived cortical neurons. In this model, macrophage cells matured into microglia, expressed microglia-specific markers such as P2Y purinoreceptor 12 (P2RY12) and MER proto-oncogene tyrosine kinase (MERTK), portrayed their phagocytic function, and were ramified (Haenseler et al., 2017). It was also observed that the iPSC-derived microglia were able to become activated, taking on an amoeboid morphology and releasing inflammatory products. The co-culture was stable for many weeks and microglia expressed many genes associated with AD, such as *TREM2, APOE, APP*, and *PARK15* (Haenseler et al., 2017), making this an effective tool for studying microglia in disease.

Using the opportunity to access brain tissue at the time of the post-mortem examination, isolated microglia expressed constitutive microglial markers such as CD11b, CD32, CD64, CD68, and HLA-DR. Cells sourced from AD brains also presented elevated pro-inflammatory molecules including cytokines interleukin 6 (IL6), tumor necrosis factor-alpha (TNFα), CCL3, CCL4, CXCL8, and M-CSF (macrophage colony-stimulating factor) suggesting a greater inflammatory response from microglia in AD (Lue et al., 2001). A study showed that it was possible to distinguish microglia (CD11b^++^CD45^dim^) from macrophages (CD11b^++^CD45^high^) using flow cytometry after extraction from *post-mortem* brain tissue (Melief et al., 2012). Upon LPS stimulation, microglia responded by upregulating inflammatory markers (Melief et al., 2012) as seen *in vivo* after ‘injury’ occurring in the human brain (Zrzavy et al., 2019). Further analysis of this isolation method has shown the technique to be extremely efficient, yielding up to 450,000 cells per gram of brain tissue (Mizee et al., 2017). Examining the role microglia play in the spread of tau in AD, primary human microglia were isolated from *post-mortem* disease tissue and analyzed for tau seeds. The study reported that Tau seeds were form of misfolded tau protein and were thought to be the template from which the spread of pathological tau occurs. Interestingly, the isolated microglia contained the tau seeds and were able to further release them into the surrounding media. This suggests that microglia could uptake the tau via phagocytosis with the aim to remove the toxic protein; however, this process does not appear to be sufficient to neutralize the tau seed (Hopp et al., 2018).

It is assumed that as the cells are sourced directly from a donor brain, they may better recapitulate the characteristics of human microglia *in vivo* than microglia isolated from a rodent brain or human iPSCs cultures. However, this process needs to be improved as the microglial-specific markers were observed to diminish over time in culture (Mizee et al., 2017), making the technique less effective for long-term use without stability improvements and thus less attractive for the study of chronic neurodegenerative conditions.

### Astrocytic Cell Culture Models

Astrocytes can also be successfully derived from human iPSCs, through intermediate-stage neural progenitor cells (NPCs) (Shaltouki et al., 2013; Tcw et al., 2017) or oligodendrocyte progenitor cells (Jiang et al., 2013). Similarly to microglia, astrocytes can be derived from healthy individuals’ iPSCs or generated from patients with known genetic mutations to understand familial diseases.

Deriving astrocytes from the iPSCs of a patient with the PSEN1 M146L familial AD mutation created cells that expressed normal astrocyte markers GFAP, S100B, EAAT1, and Glutamine Synthetase (although cellular localization of the former three markers was altered). These cells were atrophied, with lower surface area and volume than those from the control donor. However, regardless of the presence or absence of the PSEN1 M146L genotype, iPSC-derived astrocytes expressed the chemokines CXCL8 and CCL2, as well as tissue inhibitor of metalloproteinases 2 (TIMP-2) (Jones et al., 2017). The effect of APOE genotypes on iPSC-derived astrocytes was also investigated, with the APOE ε4/ε4 astrocytes showing similar morphologies and expression of the same astrocyte markers and chemokines (with the addition of the upregulation of CCL4 and CCL5) to the PSEN1 M146L astrocytes (Jones et al., 2017). APOE ε4/ε4 astrocytes also showed evidence of increased cholesterol synthesis and release and had impaired clearance of oligomeric Aβ_42_ (Lin et al., 2018), and were less effective in supporting neurons as illustrated by lower levels of synaptic proteins in the co-culture (Zhao et al., 2017). Exacerbated cytokine expression was also shown in another iPSC study (from PSEN1 ΔE9 mutation patients) which also noted increased Aβ pathology and Ca^2+^ release from the endoplasmic reticulum of mutant cells (Oksanen et al., 2017). These results may suggest an astrocyte contribution to AD through altered inflammatory protein expression associated with an atrophic morphology.

To understand the activity of human astrocytes, iPSC-derived cells were cultured under different conditions using signaling factors to modify the astrocytes phenotypes to either mature or reactive cells observed *in vivo*. Incubation with TNFα led to increased expression of CXCL8, CCL5, and lipocalin 2 (LCN2) (Roybon et al., 2013), and co-stimulation with TNFα and IL1β increased CXCL8, CCL5, and LCN2, as well as Complement C3 (a marker of A1 astrocytes) and IL6 (Hyvärinen et al., 2019). Of note, these inflammatory molecules along with the chemokines CXCL10, CCL2, and CXCL8 were also increased in human primary fetal astrocytes after TNFα stimulation (Croitoru-Lamoury et al., 2003; Meeuwsen et al., 2003). Since TNFα is primarily released from activated microglia in the brain, it reinforces the idea that microglial activation has consequences on the astrocyte profiles. To look at astrocytic aging, cells derived from NPCs from young (0.5–3 years) and older (42–56) donors were compared. A more reactive phenotype was noted in older derived astrocytes, which had larger surface areas and showed higher intensities of two intermediate filament proteins, GFAP and vimentin (Gatto et al., 2021).

### Microglia/Astrocyte Co-cultures

To mimic the complexity of the human brain, a recent model was developed encompassing human NPCs alongside human microglia and astrocytes (Park et al., 2018). This revolutionary system used a 3D microfluidic approach which allowed the recapitulation of the cells natural microenvironment by creating chemical gradients (Halldorsson et al., 2015). For the first time *in vitro*, this 3D human tri-culture model was able to represent the kinetics of the pathophysiological mechanism of key features of AD at the different stages of the disease, with the presence of Aβ aggregation, microglial activation and tau accumulation (Park et al., 2018). The increased cytokine levels observed suggested that microglia were being activated in response to and migrated toward Aβ deposition and this was associated with neuronal/neuritic damage. In addition, neurotoxic activity, axonal cleavage and nitric oxide release were observed, all consistent with inflammatory features of human AD (Park et al., 2018).

## Post-Mortem Studies

*Post-mortem* examination remains the gold standard for the diagnosis of neurodegenerative conditions and thus has been essential in emphasizing the role of glial cells in disease. However, this type of study only provides a ‘snapshot’ rather than a temporal profile of the events, usually assessing the state of the brain at the end stage of the disease. Occasionally studies attempted to assess progression by designing cohorts with a spread of neuropathological severity. However, since obtaining *post-mortem* tissue can be problematic, this is not always possible. Another challenging aspect of working with human brains is the high number of cases required to achieve statistical power and meaningful data due to human variability. This contrasts with animal studies in which data are less varied due to the animals being genetically identical, and thus require a smaller number of animals, but they do not replicate the complexity of human diseases. This emphasizes the need for human comparison, and in the last 20 years, the establishment of brain banks has facilitated the access of human tissue to researchers (Gomez-Nicola and Boche, 2015; Hartnell et al., 2021). This has been associated with the development of antibodies specific to glial proteins, essential to the morphological and functional identification of the cells allowing qualitative and quantitative studies (Magaki et al., 2019) (Table 2). By comparing control and disease brains, the effects of disease on microglia and astrocyte number, morphology, and function have been explored.

**TABLE 2:** Most common microglia and astrocyte immunomarkers and their functions.

Microglia	Astrocyte
*Iba1* – motility and migration	*GFAP* – cytoskeletal protein
*P2Y12* – motility	*Aldh1l1* – pan astrocyte marker involved in folic acid metabolic process
*CD68* – phagocytosis	*S100B* – calcium binding protein
*TMEM119* – microglial-specific marker (unknown microglial function)	*EAAT1/2* – glutamate transporters 1 and 2
*HLA-DR* – antigen presentation	*Glutamine Synthetase* – enzyme that contributes to the metabolic regulation of glutamate
*CD64* – high affinity binder of monomeric IgG antibodies	

### Microglial Studies

**FIGURE 4:**
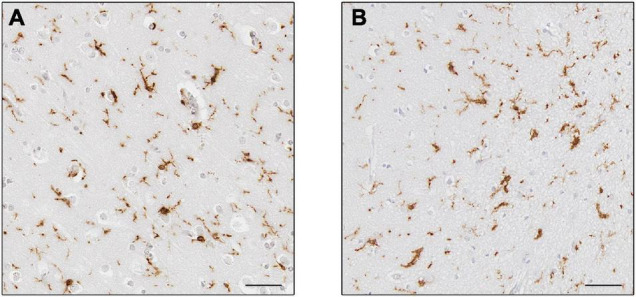
Expression of Iba1+ microglia. Microglia in controls **(A)** and Alzheimer’s disease **(B)**. Cells in **(B)** appear to have thicker processes. Scale bars = 50 μm.

Numerous *post-mortem* studies have been performed concerning microglia in AD, and thus a few recent examples will be described here to highlight our current knowledge. For example, a systematic review conducted in 2017 demonstrated that the markers CD68 and major histocompatibility complex (MHC II), acknowledged as markers of microglial activation linked to phagocytosis and antigen-presentation, respectively, were increased, while the expression of CD11b (complement receptor 3) and ionized calcium-binding adapter molecule 1 (Iba1) (Figure 4), associated to microglial motility (Franco-Bocanegra et al., 2019b) were not different between the control and AD cohorts. This was confirmed by quantification of cell counts, which stated the number of microglial cells did not differ between the cohorts, but their activation was increased (Hopperton et al., 2018). This implies that microglia were responding to the disease state by altering their functions (becoming activated) rather than by increasing their numbers. A study investigating microglial activation in the hippocampus during the course of the disease described an abnormal morphology of microglia in the late stage of AD. Microglial morphology was characterized by the presence of fragmented processes (known as dystrophic microglia), a decrease of the cell arborization and of the cell numbers associated with a decrease of the area of surveillance usually monitored by microglia. These features were observed using Iba1 and P2RY12 immunostaining and were consistent with the absence of increased expression of several microglial genes (*CD11b*, *Iba1*, *TREM2*, and *CD33*). The abnormal morphology was interpreted as microglial degeneration, highlighting the presence of dysfunctional microglia in AD, possibly as the result of the accumulation of phosphorylated tau, the dominant neuropathological hallmark in the hippocampus (Sanchez-Mejias et al., 2016). Of note, both studies reported the absence of increased cell number but the presence of different phenotypes of microglia, differences maybe due to the immunomarkers used, the brain area studied and/or the pathology presents, highlighting the heterogeneity of the microglial populations and of their responses in human AD.

Nevertheless, studying individual markers of microglial functions does not capture the variation of microglial phenotypes present in the brain, so one study used a combination of seven microglia/macrophages markers (TMEM119, CD32, CD74, CD163, CD206, P2Y12, and L-Ferritin) and the activated marker (HLA-DR) to establish the functions of microglia in non-pathological *post-mortem* brain tissue. HLA-DR was highly co-labeled with CD32, CD163, and CD206 (Swanson et al., 2020). CD32 is a Fc gamma receptor (FcγRII) involved in phagocytic capabilities of microglia (Peress et al., 1993), while CD163 (haptoglobin-hemoglobin complex receptor) and CD206 (mannose receptor) (Galea et al., 2005) are associated with the alternate activation profile (Walker and Lue, 2015). This study showed that microglia possess high heterogeneity in *post-mortem* tissue, based on the high vs. low expression as done in flow cytometer cell sorting. They detected different functional groups including a microglial population defined as Iba1^low^/L-Ferritin^high^ associated with CD74 and HLA-DR expression and defined as dysfunctional based on its overrepresentation in AD, consistent with single-cell transcriptomic studies (Swanson et al., 2020). The use of myeloid markers means that the findings were not able to definitively be accounted for by microglia only, implying the involvement of perivascular macrophages. Indeed, as mentioned by the authors in their study (Swanson et al., 2020), CD206 is specifically expressed by perivascular macrophages (De Picker et al., 2021; Shtaya et al., 2021).

Another study examined the levels of Translocator Protein (TSPO) in *post-mortem* brains. TSPO has been identified as a positron emission tomography (PET) ligand to assess microglial activation in patients *in vivo* (discussed further in the imaging section) (Werry et al., 2019). Immunodetection of TSPO showed that AD patients had significantly higher expression of the protein, consistent with the increased signal observed in patients, and interpreted as an increased amount of activated microglia in AD. However, they also reported astrocytes, endothelial cells and smooth muscle cells positive for TSPO staining and an absence of a significant difference in TSPO expression between controls and AD. Therefore, it was concluded that the TSPO function remains to be fully understood (Gui et al., 2020).

A large-scale *post-mortem* study was conducted on 299 cases, where brain tissue was immunohistochemically analyzed for different microglial phenotypic markers such as CD68 (phagocytosis), HLA-DR (antigen presentation), Iba1 (microglia motility), methionine sulfoxide reductase (MSR-A – phagocytic marker specific to Aβ) and CD64 (FcγRI with high affinity for immunoglobulins). The study revealed that CD68, MSR-A, and CD64 were increased in relation to cognitive decline in AD, whereas Iba1 was decreased in these brains (Minett et al., 2016). This indicated that phagocytic function was increased with worsening AD, highlighting the microglial response to neurodegeneration. Of note, the opposite relationship with Iba1 and cognition indicated that microglia were less motile and thus less able to support neurons and potentially to respond to a further insult as the disease might be developed. Furthermore, the APOE ε4 (risk) allele was associated with CD68, HLA-DR, and CD64 expression, suggesting an exacerbated microglial response in presence of ε4. In contrast, the protective APOE ε2 allele was associated with microglial motility (Iba1), essential to the physiological function of microglia (Minett et al., 2016). This study highlighted the importance of microglial motility in the response to homeostatic changes in keeping the brain healthy. Using the same cohort, TREM2 immunostaining was performed and interestingly, monocytes rather than microglia or perivascular macrophages were immuno-labeled. This was in contrast with the findings in experimental models and may highlight another difference between rodents and human microglia (Fahrenhold et al., 2018).

A limitation of human *post-mortem* studies compared to animal models is the difficulty investigating changes in microglia after disease manipulation. Therefore, access to *post-mortem* tissue from patients involved in clinical trials should be a priority. Indeed, access to the brains of AD patients immunized against Aβ has allowed unique observations. For example, exploration of microglial motility was performed using Iba1, P2Y12, CFL1, and Coronin-1A (CORO1A) markers in association with Aβ and tau (Franco-Bocanegra et al., 2019b). No difference in motility markers was detected between controls and AD cases; while in immunized AD cases, the homeostatic markers Iba1 and P2Y12 were both increased. Interestingly, Aβ was associated with Iba1 and P2Y12 in controls, but this association was lost in AD and not restored in the immunized AD cases. This suggested that microglia respond to Aβ deposition in the healthy brain but then the cells became dysfunctional in AD and that immunotherapy did not restore the physiological conditions, despite the increased expression of both markers (Franco-Bocanegra et al., 2019b).

In the last 5 years, numerous microglial markers have been developed as heterogeneity of microglia within the same brain has emerged from transcriptomics and animal models. The development of novel methodologies to use *post-mortem* tissue such as multiplex staining will bring key information on glial cells in humans. A study investigating human microglia heterogeneity used multiplex staining of 57 different markers to determine if microglia within the same brain can have different phenotypes and whether they were distinguishable from other mononuclear cells. Microglia obtained from healthy donor brains had a distinct phenotype from other myeloid cells, with TMEM119 and P2Y12 being only and highly expressed by microglia (Böttcher et al., 2019). Furthermore, using multiplexed single-cell mass cytometry, microglia from the sub-ventricular zone and thalamus of *post-mortem* brains had a marked difference in phenotype compared to microglia from other brain regions, with an increase in expression of CD11c, CD45, CD64, CD68, CD195, CX3CR1, and HLA-DR, profile that they proposed reflected an activated microglia phenotype (Böttcher et al., 2019). This re-emphasized the heterogeneity of microglia within the same brain in humans.

### Astrocyte Studies

Several astrocytic proteins are considered constitutive markers of astrocytes and are used to detect these cells in human tissue. The most commonly used marker is GFAP, a major component of the intermediate filament of the astrocyte cytoskeleton. As such, it highlights extensive astrocytic processes (Waller et al., 2016) throughout the cortex, staining all four subtypes of astrocytes in the human brain (Oberheim et al., 2009) (Figure 2). The increased expression of GFAP is a feature of several neurodegenerative disorders (Pelvig et al., 2003; Schofield et al., 2003) and is thought to relate to reactive astrocytosis (Escartin et al., 2021). However, it is contested as to whether this is the consequence of increased astrocyte number or increased GFAP expression in individual astrocytes (Perez-Nievas and Serrano-Pozo, 2018). S100B and aldehyde dehydrogenase (Aldh1l1) are astrocytic markers restricted to the cell body in humans with the latter preferentially expressed in gray matter astrocytes (Waller et al., 2016). Aldh1l1 and GFAP were increased in AD, but in patients with dementia with Lewy bodies (DLB), only an Aldh1l1 increase was detected (Garcia-Esparcia et al., 2018), highlighting different pathological mechanisms between the 2 most commons types of dementia (rather than a common mechanism) with a different astrocytic contribution.

Functional markers are now often used, mostly related to glutamate processing. EAAT1 and 2 are excitatory amino acid transporters responsible for the uptake of glutamate from the synapse into astrocytes for cycling. These markers stain extensive process trees of stellate astrocyte in the gray matter. Glutamine synthetase is an enzyme in astrocytes that breaks down glutamate into glutamine after uptake through the EAATs. It primarily stains the astrocyte cell bodies but may also stain the somatic end of the processes (Waller et al., 2016). Astrocytic glutamate cycling may play an important role in mediating the disconnection between pathology and dementia symptoms. It has been shown that cognitively normal patients with AD pathology have astrocytes with longer and thicker processes than those with dementia (Kobayashi et al., 2018). Importantly, as well as showing neurodegeneration, AD pathology patients with dementia symptoms also show a reduction in EAAT2 expression (Kobayashi et al., 2018).

Finally, as activated astrocytes possibly exist with neuroprotective and neurotoxic phenotypes, it is important to detect cells of these phenotypes with specific. As C3 was upregulated in only A1 astrocytes (Liddelow et al., 2017), it has been assumed to be a marker of this phenotype, although this simplification is now disputed (Escartin et al., 2021). Nevertheless, the presence of C3^+^/S100β^+^ cells was increased in several neurodegenerative diseases including HD, AD, MND, and PD (Liddelow et al., 2017). Another marker, guanylate binding protein 2 (GBP2) is also considered to be indicative of A1 astrocytes. Whilst both markers showed an increase in Creutzfeldt-Jakob disease (CJD) cases, a disease associated with severe astrogliosis, in controls, C3 displayed high non-specific background staining in human tissue, whereas and GBP2 was highly abundant in GFAP+ astrocytes in disease tissue and was completely absent in control tissue (Hartmann et al., 2019). The C3 staining highlights the challenges in staining diffusible proteins compared to proteins located in the cell compartment or at the cell membrane, thus GBP2 seems to be more suitable to identify polarized A1 astrocytes in humans. Of note, the relationship between constitutive and functional markers is important to consider as situations exist where levels of the EAAT2 marker were unaltered, yet the levels of a constitutive maker (Aldh1l1) were changed (Garcia-Esparcia et al., 2018), which may actually represent the proliferation of astrocytes, yet a downregulation of EAAT2 in each astrocyte. Thus, functional alterations in astrocytes may be present in the condition but missed when viewing markers independently.

## Imaging Studies

The most challenging aspect in the study of microglia and astrocytes is that the cells are not visible during life. iPSC-derived cells are taken out of the system in which they act and are usually derived from non-cerebral cells, and cells studied *post-mortem* are not dynamic. Therefore, methods have been developed to non-invasively assess microglia and astrocyte activity in the human brain through imaging techniques. This can be done via PET imaging that involves the administration of a radioactive PET ligand to patients which will cross the blood–brain barrier and recognize specific receptors in the brain (Boche et al., 2019). These radioactive ligands release gamma rays that are detected by gamma cameras to show the signal of receptor-bound ligands in 3-dimensions. These ligands bind to receptors whose expression is related to the activation of microglia and astrocytes and this can show the neuroinflammatory status of the brain *in vivo.* Magnetic resonance imaging (MRI) is another method in which neuroinflammation can be detected. Specialized MRI techniques can show the diffusivity of water through tissue and highlight areas of gliosis (where the flow of water is disrupted by neuroinflammation). While MRI can be a good method of visualizing neuroinflammation, PET imaging is the current gold standard as a functional imaging technique to detect the activation of specific cell types.

### Microglial Imaging

The first PET ligand to identify microglia and neuroinflammation is the translocator protein (18KDa) (TSPO) ligand, as mentioned above. Initially, this molecule was called peripheral benzodiazepine receptor (PBR) but later changed to suit its structure and function more accurately (Papadopoulos et al., 2006). There are many different TSPO ligands with their efficacy constantly improved in terms of binding capabilities and half-life. The ligand [^11^C]PK11195 was the first TSPO-PET ligand used to identify microglia with findings showing its binding increased in patients with AD compared to controls (Cagnin et al., 2001). Limitations of PK11195 impeded its use, including short half-life, a high signal to noise ratio meaning that non-specific binding may occur (Werry et al., 2019).

The DPA family of TSPO ligands is a group of second-generation PET ligands which have come to the forefront of microglia research. These are fluorine-based with a longer half-life and a better microglial specificity than the previously used carbon-based ones like PK11195 (Werry et al., 2019). However, the use of the second-generation ligands has been hampered by the binding affinity linked to the single nucleotide polymorphism (SNP) in the *TSPO* gene with no binding in the presence of the *rs6971* SNP, which affects 1/3 of Caucasians (Werry et al., 2019). Consequently, patients have to be screened for *TSPO* polymorphism and analysis of TSPO binding affinity to correct for this factor. Among these ligands, DPA-714 has shown interesting results in the time course of AD. A study showed that patients with a high initial DPA-714 binding presented a lower level of microglial activation in the later stages of the disease. Whereas, in patients with a low TSPO binding level in early AD, microglial activation was later increased and had worse disease severity (Hamelin et al., 2016). The authors proposed a protective role of microglia early in the disease.

Since, the third generation of TSPO ligands has been designed to counter the effects of the *TSPO* polymorphism, based on improving the PK111-95. The [^11^C]ER176 ligand has so far only been tested in healthy controls. This ligand has been developed based on the incorporation of fluorine-18 to increase the stability and thus facilitate the use of TSPO-ligands in the clinical environment, but further work is required to assess its benefit in a clinical environment.

Translocator Protein is not the only target for microglia PET imaging. As it is currently unknown whether TSPO shows a pro- or anti-inflammatory microglial phenotype, other ligands are being developed in order to explore the inflammatory profile of microglia. Two potential novel microglial PET ligands have been suggested: the ionotropic P2X receptor P2X7, which is expressed in microglia, macrophages and monocytes (Oliveira-Giacomelli et al., 2021), and the metabotropic P2Y receptor P2Y12, which is only expressed by microglia and considered as a homeostatic marker (Beaino et al., 2017; Mildner et al., 2017; Franco-Bocanegra et al., 2019b). Experimental studies have observed P2Y12 expressed by anti-inflammatory microglia and P2X7 by pro-inflammatory microglia. Also, binding of P2X7 PET ligand was increased in multiple sclerosis (MS) active lesions, whereas the signal of the P2Y12-PET ligand was decreased in all MS lesions (active, chronic active and chronic inactive) (Beaino et al., 2017). To our knowledge, the expressions have not been tested in AD. Cannabinoid receptor type 2 (CBR2) has been proposed as another potential target for PET tracers of microglia (Núñez et al., 2004). However, [C^11^]NE40 (a CBR2 ligand) binding was lower in AD compared to controls and there was no association between the ligand and Aβ plaques (Ahmad et al., 2016), suggesting that this may not be a good target for the identification of activated microglia in disease.

Magnetic resonance imaging has been less utilized than PET to assess gliosis due to its lack of cell specificity. Of note, it was recently suggested that iron associates with microglia in AD, especially in dystrophic microglia defined as senescent or dysfunctional (Streit et al., 2009), and microglia primed to phagocytose Aβ (Kenkhuis et al., 2021). MRI is very sensitive to iron (Zeineh et al., 2015) and therefore might be used to find brain areas with microglial populations involved in AD.

Interestingly, a study using diffusion-weighted MRI built a microstructural model of diffusion based on the ramified morphology of glial cells. The model was tested on rats under several conditions and the protocol was validated for glial cells detection via the immuno-labeling of the brain rats for microglia (Iba1) and astrocytes (GFAP). Interestingly, this novel model appears not only to be able to distinguish between microglia and astrocytes but also to assess the presence of neurodegeneration. The model was then tested on six healthy patients to evaluate reproducibility of the method as a proof-of-concept. Similar patterns of microglial cell density were observed within different regions as those reported in *post-mortem* tissue (Garcia-Hernandez et al., 2021).

Magnetic resonance imaging has been widely used to assess certain characteristics of AD, such as brain atrophy, but it remains to be perfected for a precise and fully accurate detection of activated glial cells and the development of novel mathematical models are underway.

### Astrocyte Imaging

As mentioned above TSPO has been commonly used as a target for PET imaging of activated microglia. However, studies have shown that TSPO is also expressed by other cerebral cells. In AD PET studies, TSPO was increased in macrophages, endothelial cells, vascular smooth muscle cells, and most notably reactive astrocytes (Cosenza-Nashat et al., 2009; Gui et al., 2020). Indeed TSPO appeared to be specifically upregulated in astrocytes and microglia (Pannell et al., 2020), but interestingly, its expression was not related to microglia, astrocytes, Aβ, tau neurofibrillary tangles or cortical thickness in *post-mortem* AD (Gui et al., 2020). This suggests that a TSPO-PET signal may not solely highlight microglial changes, but also changes in astrocytes.

Astrocyte specific PET tracers have been developed and most commonly target monoamine oxidase B (MAO-B), which is upregulated in activated astrocytes (Ekblom et al., 1993). A common tracer used to detect this protein is ^11^C-deuterium-L-deprenyl (^11^C-DED) with the binding increased in AD vs. controls (Santillo et al., 2011), supporting a role for these cells in the disease. This study, however, did not take into account disease severity. When looking at MCI patients with and without amyloid pathology, as well as in AD patients and controls, levels of ^11^C-DED were increased in cortical and subcortical regions of MCI patients with amyloid pathology compared to control and AD patients (Carter et al., 2012). Testing the ^11^C-DED tracer on *post-mortem* tissue also revealed increased binding in brains with tau pathology in Braak Stage I-II and reduced in brains with more severe tau pathology (Gulyás et al., 2011). Findings from both of these studies imply that astrocytes become activated early in the development of AD, but that their activation is lessened as the disease progresses, perhaps due to astrocytic cell pathology. This has also been shown in autosomal dominant AD, where a pre-symptomatic increase in ^11^C-DED was detected (Rodriguez-Vieitez et al., 2016) along with a gradual decrease in astrocytosis as Aβ deposition (measured by ^11^C-PiB) rose.

Other common targets developed for the detection of astrocytosis by PET are the type-2 imidazoline receptors (I2Rs). These receptors have previously been shown to be increased in density in the brains of AD patients (Ruiz et al., 1993). The tracer ^11^C-BU99008 is a highly specific tracer for activated astrocytes in the human brain (Tyacke et al., 2018) and its binding was increased in MCI and AD patients in tandem with a decrease in glucose metabolism (Fan et al., 2018). In PD, the pattern of astrocyte activation appeared to be similar to that shown by ^11^C-DED in AD. There was an initial increase in ^11^C-BU99008 detected in all cortical regions as well as in the brainstem in early disease patients, but reductions compared to control were found in moderate/advanced disease (Wilson et al., 2019), re-enforcing the idea of astrocytes becoming dysfunctional as the disease progresses. The mechanism behind the decline in astrocytosis with disease severity is still unclear, but a strong correlation between ^11^C-DED and ^18^F-fluorodeoxyglucose (^18^F-FDG) a marker of glucose metabolism has been observed (Carter et al., 2019), suggesting that the reduced metabolism in disease may be related to astrocyte degeneration (Boche et al., 2019).

Magnetic resonance imaging mean water diffusivity (MD) as measured using diffusion tensor imaging (DTI) may be useful to non-invasively monitor neuroinflammation. In familial AD, a negative association between MRI-MD and PET ^11^C-DED signal was observed, suggesting a two-phase process, in which neuroinflammation increases cortical thickness and reduces MD through glial swelling, before the onset of neurodegeneration thinning the cortex and increasing MD (Vilaplana et al., 2020). Another specialized MRI measure of diffusivity, known as diffusion basis spectrum imaging (DBSI), is suggested to be representative of neuroinflammation. Using this technique, it was shown that white matter diffusivity decreased with increased tau and Aβ_42_ levels. This was interpreted as increased white matter inflammation in response to amyloid pathology and neurodegeneration (Wang et al., 2019), consistent with astrocytes being more numerous in white matter than gray matter and the concept of different astrocyte populations.

Another non-invasive technique called proton magnetic resonance spectroscopy (H-MRS) is useful to monitor astrocyte function *in vivo.* H-MRS detects molecules, such as metabolites and neurotransmitters, based on the unique proton and electron signature of each molecule (Ford and Crewther, 2016). This allows the monitoring of molecules involved in astrocytic metabolism as they change during aging. Using this method, it was found that in elderly patients, glutathione, an antioxidant released by astrocytes to protect neurons (Wang and Cynader, 2000), was reduced – diminishing its neuroprotection (Emir et al., 2011). Glutamate cycling components in astrocytes have also been shown by H-MRS to be altered during development and aging. Glutamate itself was detected at lower concentrations in older brains, related to the loss of neurons (Kaiser et al., 2005).

Each of these different techniques of brain imaging brings complementary information regarding the behavior of microglia and astrocytes in alive patients. To get the fullest picture of neuroinflammation *in vivo* it would be informative to combine, if possible, microglia and astrocytes PET ligands.

## Biomarker Studies

Biomarkers are often used to screen the neuroinflammatory profile of the CNS. A common compartment used is the cerebrospinal fluid (CSF), due to its relative accessibility. The CSF is usually considered to mirror brain parenchyma; however, this is debated. Plasma biomarkers have been developed but their sensitivity makes them less reliable than the CSF biomarkers. In AD, many biomarkers have been optimized based on neuropathology including Aβ_42_, total and phosphorylated tau. Recently, the focus has been on glial cells with the assessment of several inflammatory markers in the blood or CSF to identify a neuroinflammatory profile that could be used as a predictor of AD, part of the AD diagnosis or to follow therapeutic effects.

### Microglial Biomarkers

Several CSF biomarkers have been measured based on genetics or related to microglial functions. One study showed that CSF levels of TREM2 and CCL2 were positively associated with AD. Both inflammatory compounds were also increased, albeit to a lesser extent, in MCI patients, indicating that microglial activation occurs early on and increases as the disease progresses. The same study also observed an association between total tau and the microglial markers indicating that microglial activation was associated with tau rather than Aβ pathology in AD (Nordengen et al., 2019). Another study on CSF TREM2, reported higher levels of TREM2 in patients with dementia associated with a slower increase of Aβ and lower tau levels (Ewers et al., 2020), thus suggesting an initial neuroprotective effect of TREM2-positive microglia. Of note, as the expression of TREM2 by microglia remains debatable, with evidence suggesting that this marker identifies monocytes rather than microglia (Fahrenhold et al., 2018), with a role for the peripheral TREM2-positive monocytes/macrophages in AD and therefore the role of the systemic immune system in the development of AD cannot be excluded (Rakic et al., 2018).

CD14 is a cofactor for toll-like receptors (TLRs) expressed by macrophages and microglia that recognizes foreign bodies in the CNS and induces the production of pro- and anti-inflammatory cytokines (Pase et al., 2020). A mouse model knock-out for CD14 showed the presence of a decreased number of activated microglia and Aβ plaques (Reed-Geaghan et al., 2010), highlighting the potential involvement of CD14 in the inflammatory response in AD. In humans, CD14 levels were increased in the CSF of patients with AD and PD (Yin et al., 2009). In a larger cohort, CD14 was measured in plasma samples taken at baseline and compared to samples collected during a 10-year follow-up. The study found that increased CD14 baseline measurement gave a 12% increased risk of developing dementia (Pase et al., 2020). Furthermore, higher levels of CD14 were associated with MRI markers of increased brain atrophy and cognitive decline (Pase et al., 2020). However, it is not understood how the expression of CD14 in plasma correlates with its expression in the CNS. Thus, further research is required to assess whether CD14 may be an effective indicator of neurodegenerative disease.

As microglia are the primary immune cell in the brain and release pro- and anti-inflammatory cytokines, the cells represent a good target for CSF biomarkers with IL1α a good candidate. Indeed, its expression has been shown related to amyloid plaques in MCI patients (Wang et al., 2015), increased in the CSF of MCI patients and associated with the cognitive decline as measured by the mini-mental state examination (MMSE) scores (Hu et al., 2010). The pro-inflammatory cytokine TNFα is another candidate as a potential biomarker as it is increased in CSF levels in AD patients (Llano et al., 2012) and is associated with increased cognitive decline in AD patients with systemic infection (Holmes et al., 2009). Consequently, injection of etanercept, a TNFα inhibitor, tested in a randomized double-blind phase 2 trial in 40 patients with mild to moderate AD showed promising trends and was well-tolerated (Butchart et al., 2015).

### Astrocyte Biomarkers

Cerebrospinal fluid biomarkers are also useful to assess astrocyte function during the course of disease in humans. YKL40 (also named Chitinase-3-like 1) is a glycoprotein, known to be secreted by astrocytes, which has been used as a biomarker of astrocyte activation. It was found elevated in the CSF of patients with CJD and AD (Llorens et al., 2017), progressive supranuclear palsy, corticobasal degeneration, and Pick’s Disease (Querol-Vilaseca et al., 2017), but not in vascular dementia or PD. Of note, there is debate as to the expression of YKL-40 in microglia, with studies suggesting that the high levels seen in microglia *in vitro* are not matched *in vivo* (Bonneh-Barkay et al., 2012). Both previous CSF studies, however, confirmed the astrocytic expression of YKL40 and noted no colocalization with microglia or neurons. Regardless of whether microglial YKL40 expression contributes to the CSF levels, microglia are involved in the induction of expression of YKL40 from astrocytes through the release of IL1β and TNFα (Bonneh-Barkay et al., 2012). Interestingly, YKL40 has been classified as an anti-inflammatory protein (Rakic et al., 2018).

Being the most commonly used astrocyte marker, GFAP may also be useful as a CSF biomarker of astrocyte activity. It is detectible in the CSF of AD patients and is correlated with the cognitive score assessed with the MMSE (Fukuyama et al., 2001). It was also detected in the blood plasma of patients with AD with GFAP being significantly increased in patients with a positive amyloid PET status compared to those with negative amyloid PET results (Thijssen et al., 2020). As the astrocytic marker S100B also showed increases in early (Peskind et al., 2001) and more advanced AD (Petzold et al., 2003), whilst findings related to glutamine synthetase have been conflicting (Tumani et al., 1999; Timmer et al., 2015).

The use of both biomarkers such as CSF markers and neuroimaging markers (PET and MRI markers) in conjunction with each other could provide a wider view of the microglial profile in the brain and give better insight into the role these cells play in neurodegeneration and disease.

## Microglia and Astrocyte Communication

**FIGURE 5:**
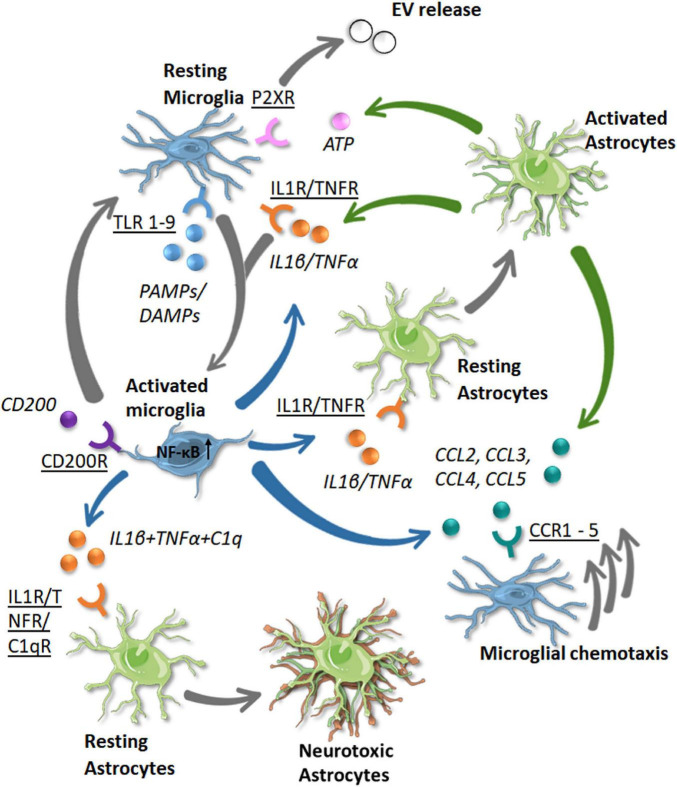
Known communication pathway between microglia and astrocytes in the human brain in response to disease. Receptors (underlined) and the signaling molecules (italic) are shown with the color of the arrow denoting the cell source (green = astrocytes, blue = microglia). The resulting phenotypic changes or movements are shown by gray arrows (single or triple, respectively). As microglia become activated by PAMP/DAMP signaling via TLR receptors (both blue), they become activated (gray arrow). Activated microglia release IL1β and TNFα which bind through IL1R and TNFR receptors to activate astrocytes and other microglial cells. This signaling with the addition of C1q signaling on astrocytes leads to a neurotoxic phenotype, with a loss of neuroprotective functions. Activated astrocytes release IL1β and TNFα to propagate a neuroinflammatory response. They also release ATP which binds to P2XR receptors on microglia, leading to extracellular vesicle release. Activated astrocytes and microglia may also release chemokines (CCL2–CCL5) which bind to receptors on microglia (CCR1–CCR5) and stimulate their chemotaxis toward the site of injury/pathogen. Inflammation is in part resolved by CD200 binding to CD200R on activated microglia, leading them to return to a homeostatic phenotype.

Microglia and astrocytes are key cells of the CNS, communicating between themselves to coordinate their activities as well as with the neurons to support them. The most studied interactions between microglia and astrocytes in humans have been investigated in pathological conditions where microglia and astrocytes respond to the homeostatic changes, becoming activated and altering their phenotype to enact a response. At the initiation of this cascade of activation in the context of AD, it is thought that pattern recognition receptors (PRRs) such as TLRs are involved. Eleven TLRs are expressed in humans (Kielian, 2006) with each recognizing specific pathogens and/or damage activated molecular patterns (DAMPS/PAMPS). Microglia robustly express a wide range of TLRs (Kielian, 2006) (Figure 5); whereas astrocytes preferentially express TLR3 receptors, with low TLR1, TLR4, TLR5, and TLR9 expression, and absent expression of TLR2, TLR6, TLR7, TLR8, and TLR10 (Jack et al., 2005). One such ligand used experimentally is lipopolysaccharide (LPS) which is expressed on the cell wall of Gram-negative bacteria and binds to TLR4. The low level of TLR4 expression in human astrocytes (Bsibsi et al., 2002; Jack et al., 2005) may account for their lack of response to LPS stimulation (Peterson et al., 1997). Another study using human cultured cells (collected *post-mortem*) found that whilst microglia expressed mRNA of TLRs 1–9, astrocytes only show robust expression of TLR2 and TLR3 (Bsibsi et al., 2002). This comparatively low expression of TLRs in astrocytes may imply that they cannot directly respond to many pathogens and require microglia to detect the pathogen and signal to astrocytes to induce activation. This idea is in line with two studies which showed that after LPS activation, only microglia and not astrocytes release β-chemokines CCL2 (MCP-1), CCL3 (MIP-1α), CCL4 (MIP-1β) (Peterson et al., 1997) and CCL5 (RANTES) (Hu et al., 1999). Both studies showed that upon cytokine activation (with IL1β or TNFα) but not LPS stimulation, astrocytes were capable of releasing chemokines, suggesting that astrocytes were incapable of responding to LPS. All of this suggests that microglia may be more sensitive to pathogens than astrocytes and the likely pattern of glial activation first requires microglial activation in response to the pathogen before the signal of activation is propagated to astrocytes via inflammatory cytokines.

As well as being activated, these cells also need to migrate to the location of the insult. β-chemokines (mentioned above) are important chemoattractant molecules that mediate cell motility. As well as differences in their release of chemokines, microglia and astrocytes also have different motile responses. Indeed, CCL2, CCL3, and CCL4 added to media in chemotaxis chambers lead to the migration of human microglia but not astrocytes (Peterson et al., 1997). However, a contradictory study shows that CCR2 [the receptor for CCL2 known to be expressed by microglia (van der Meer et al., 2000)] was detected in human fetal astrocytes and mediated their chemotaxis (Andjelkovic et al., 2002).

Changes to chemokine levels may have relevance to neurodegenerative diseases with plasma CCL2 increased in MCI and AD patients (Galimberti et al., 2006; Lee et al., 2018) and correlated to memory decline (Bettcher et al., 2016, 2019). CCL4 expression was enriched in reactive astrocytes in AD brains (Xia et al., 1998). However, due to the lack of a chemotactic response in human astrocytes, astrocytes may use CCL4 to signal to microglia, although it is unclear whether CCL4 has any other effect. As both human microglia and astrocytes are known to release IL1β and TNFα (Hanisch, 2002; Choi et al., 2014) (Figure 5), it seems that both cells are capable of propagating the activation signal to other glial cells to induce their chemotaxis.

The propagation of the inflammatory signal to astrocytes may not always be beneficial to the CNS environment, with exacerbated activation contributing to neurodegeneration rather than protecting against it. Indeed, the neurotoxic phenotype (A1) is a result of cytokine signals (TNFα + IL1α + C1q) released from activated microglia (Figure 5). Once this occurs, A1 astrocytes lose their core functions of phagocytosis and are unable to aid in neuronal survival. Whilst each of IL1α, TNFα, and C1q stimulate the majority of genes in the A1 transcription profile, the three cytokines are required to fully recapitulate the A1 transcriptome (Liddelow et al., 2017), as in human iPSC astrocytes (Barbar et al., 2020). The A1 profile was related to reductions in neuronal support functions such as reduced glutamate uptake and reduced phagocytosis. This shows another example of how microglia-astrocyte communication plays an important role in the brain, even in a negative way with A1 astrocyte activation by microglia contributing to the worsening of neurodegenerative disease.

Microglia and astrocytes need to have a bidirectional communication of inflammatory signals for efficient immune response in the CNS. However, it is also important that there is a ‘resetting’ mechanism for microglia back to their physiological state. When microglia are in their homeostatic state, a molecule known as CD200 is responsible for crosstalk between microglia and other cells such as astrocytes and neurons. CD200 is a glycoprotein expressed by many cells in the brain, but its receptor, the CD200R, is only found on microglia and macrophages. This makes the communication between microglia and other cell types one-sided in this instance. When CD200 interacts with its receptor on microglia, this instates the surveying/physiological state (Szepesi et al., 2018) (Figure 5). Under normal conditions, astrocytes do not express CD200, though its expression was found to colocalize with GFAP in reactive astrocytes in multiple sclerosis (Koning et al., 2009). It was observed that disturbances in CD200 signaling occur in AD, with a decrease in the protein in areas of the brain predominantly affected by the disease (Walker et al., 2009). This could suggest that the inability of cells such as astrocytes to communicate an anti-inflammatory signal to microglia may be a significant problem in AD and could be a potential therapeutic target of inflammation.

Another mechanism in which astrocyte and microglia communication is performed is through cholesterol, an important factor of AD with ApoE (a cholesterol transport protein) being the most important risk factor. Astrocytes are the primary cell type responsible for the synthesis of cholesterol in the brain (Pfrieger and Ungerer, 2011). Cholesterol levels are relatively low in the brain in adulthood (Lavrnja et al., 2017), compared to development where it is needed for the creation of myelin. However, cholesterol remains an important molecule in maintaining microglia as observed *ex vivo* (Bohlen et al., 2017; Goshi et al., 2020), possibly as part of the cell membrane maintenance. Additionally, cholesterol is an important factor in the inflammatory microenvironment of the CNS. Its accumulation can lead to the disruption of the phagocytic ability of microglia. Also relevant to AD is that microglia derived from patients with an APOE ε4/ε4 genotype show intracellular and extracellular cholesterol accumulation (Lin et al., 2018). A study reported that in human iPSC co-culture derived from AD brains expressing the risk variant APOE ε4, the astrocytic cells had abnormal cholesterol production capabilities with an increased amount of stored lysosomal cholesterol but a decrease in cholesterol released to microglia (TCW et al., 2019). Due to the nature of microglia requiring cholesterol to maintain homeostasis (Bohlen et al., 2017), this is an important finding. Astrocytes in culture also produced more proinflammatory cytokines which in turn will affect the brain environment (TCW et al., 2019). These studies suggest that the astrocyte/microglial cholesterol metabolism in the brain could be a key feature of neurodegenerative disorders.

Communication via extracellular vesicles (EVs) has been more recently established as a key process for cells in the CNS. They can act as a signaling complex and also allow the transport of transfer mRNA and other molecules that modulate cell function (Basso and Bonetto, 2016). EVs are important mediators of microglia-astrocyte crosstalk as they can be released and taken-up by both cell types (Budnik et al., 2016; Dickens et al., 2017). Microglia release EVs when their purinergic receptors (P2X7) come into contact with high levels of ATP released from astrocytes (Bianco et al., 2005) (Figure 5). These EVs have been shown to be released from human primary astrocytes under normal conditions and with IL1β treatment (You et al., 2020) and from microglia TNFα or non-activated microglia (van den Broek et al., 2020). When a neuronal injury occurs, astrocytes and microglia communicate to release microglial EVs containing IL1β in order to survey the parenchyma and to start an inflammatory response if needed to respond to pathogens (Budnik et al., 2016).

To investigate the role of microglia/astrocyte communication in the clearance of protein deposition, human iPSC-derived microglia and astrocytes were co-cultured in the presence of Aβ or α-synuclein (αSYN) fibrils. Firstly, it was observed that microglia and astrocytes in co-culture had a greater ability to clear the protein aggregates compared to single-cell cultures, Rostami et al. (2021). This indicated synergy between the two cell types to provide a better mechanism of clearance. Moreover, live imaging analysis from the co-culture showed the appearance of novel physical structures called nanotubes (tunneling Structures made from cell membranes) keeping the astrocytes and microglia in direct contact for effective communication (Rostami et al., 2021). A previous study conducted by the same group gave evidence that astrocytes were able to link via these structures to other astrocytes (Rostami et al., 2017). This study confirms the existence of similar structures that can also physically attach microglia to astrocytes. These nanotubes allowed for intracellular αSYN to be passed from one cell to another, in order for the fibrils to be broken down and removed from the culture. Interestingly, when this phenomenon occurred, the microglia were the cells that would attract and clear the intracellular protein from the astrocytes, not the other way around (Rostami et al., 2021), implying that while astrocytes were capable of clearing pathological proteins, microglia were the primary cells to induce degradation.

## Conclusion

The importance of microglia/astrocyte crosstalk has recently emerged as an important component of the neurodegenerative aspect of AD. However, our current knowledge of microglia/astrocyte communication is based on findings obtained from experimental animal models. These models are important as they allow the investigation and manipulation of cells and cellular interactions within a complete living system. However, to be relevant to the human condition, and particularly in relation to human disease, the findings need to be confirmed in humans. Whilst the investigation of active microglia/astrocyte communication may be challenging *in vivo*, its consequences may be measured by the presence of specific inflammatory compounds in the CSF and blood samples as biomarkers or in the brain using imaging techniques. Studies investigating dynamic communication in human cells involve primary or iPSC culture methods, which are perhaps the most useful tool in confirming functions and phenotypes of these cells in humans. Consequently, several pathways of communication have emerged that merit further exploration in homeostasis and disease conditions.

## Author Contributions

All authors listed have made a substantial, direct, and intellectual contribution to the work, and approved it for publication.

## Conflict of Interest

The authors declare that the research was conducted in the absence of any commercial or financial relationships that could be construed as a potential conflict of interest.

## Publisher’s Note

All claims expressed in this article are solely those of the authors and do not necessarily represent those of their affiliated organizations, or those of the publisher, the editors and the reviewers. Any product that may be evaluated in this article, or claim that may be made by its manufacturer, is not guaranteed or endorsed by the publisher.
